# Metabolic markers in relation to hypoxia; staining patterns and colocalization of pimonidazole, HIF-1α, CAIX, LDH-5, GLUT-1, MCT1 and MCT4

**DOI:** 10.1186/1471-2407-11-167

**Published:** 2011-05-12

**Authors:** Saskia E Rademakers, Jasper Lok, Albert J van der Kogel, Johan Bussink, Johannes HAM Kaanders

**Affiliations:** 1 Department of Radiation Oncology, 874 Radboud University Nijmegen Medical Centre, P.O. Box 9101, Nijmegen 6500 HB, The Netherlands

## Abstract

**Background:**

The cellular response of malignant tumors to hypoxia is diverse. Several important endogenous metabolic markers are upregulated under hypoxic conditions. We examined the staining patterns and co-expression of HIF-1α, CAIX, LDH-5, GLUT-1, MCT1 and MCT4 with the exogenous hypoxic cell marker pimonidazole and the association of marker expression with clinicopathological characteristics.

**Methods:**

20 biopsies of advanced head and neck carcinomas were immunohistochemically stained and analyzed. All patients were given the hypoxia marker pimonidazole intravenously 2 h prior to biopsy taking. The tumor area positive for each marker, the colocalization of the different markers and the distribution of the markers in relation to the blood vessels were assessed by semiautomatic quantitative analysis.

**Results:**

MCT1 staining was present in hypoxic (pimonidazole stained) as well as non-hypoxic areas in almost equal amounts. MCT1 expression showed a significant overall correlation (r = 0.75, p < 0.001) and strong spatial relationship with CAIX. LDH-5 showed the strongest correlation with pimonidazole (r = 0.66, p = 0.002). MCT4 and GLUT-1 demonstrated a typical diffusion-limited hypoxic pattern and showed a high degree of colocalization. Both MCT4 and CAIX showed a higher expression in the primary tumor in node positive patients (p = 0.09 both).

**Conclusions:**

Colocalization and staining patterns of metabolic and hypoxia-related proteins provides valuable additional information over single protein analyses and can improve the understanding of their functions and environmental influences.

## Background

Malignant tumors often exhibit an altered metabolism compared to normal tissues. This phenomenon can be explained by several underlying mechanisms. First of all, the genetic changes related to a high proliferation rate, as observed in many tumors, lead to an increased metabolism[1]. Another important reason for a changed metabolism is the adaptation of tumor cells to the microenvironment. Due to rapid tumor growth, hypoxic areas are frequently encountered. Under circumstances of severe hypoxia, cells are forced to use anaerobic glycolysis as their primary energy source, the Pasteur effect[2]. Normal cells convert to oxidative phosphorylation when oxygen levels are restored. In contrast, tumor cells can use aerobic glycolysis even in the presence of sufficient amounts of oxygen. This is called the Warburg effect, a manifestation of a modification of the tumor cell metabolism[3]. Due to a high level of aerobic glycolysis, in many tumor cells, glucose consumption is substantially higher than in normal cells [4,5].

The consequence of the high rate of glycolysis in malignant cells is the production of large amounts of lactic acid. An interesting observation made by Sonveaux et al. is the preference of tumor cells for lactic acid over glucose as the primary energy source [6]. This creates the perfect conditions for a symbiosis between anaerobic glycolytic cells and aerobic tumor cells [6] or aerobic stromal cells, as described in colorectal carcinomas [7].

Recently, monocarboxylate transporters (MCT's) have been discovered to play an important role in this symbiosis. These transporters facilitate the uptake and excretion of monocarboxylates, like lactate and pyruvate, and act as monocarboxylate-proton symporters[8]. MCT4 is a low-affinity/high capacity lactate transporter, which is abundantly present in highly glycolytic muscle cells. It is one of the many target genes of hypoxia-inducible factor 1 (HIF-1)[9]. MCT1 is a high-affinity, low capacity monocarboxylate transporter, found in normal tissues like the intestinal epithelium (executing an important role in organic acid absorption), the blood brain barrier, red blood cells and skeletal muscle cells. Its expression seems to be regulated by multiple signaling pathways, microenvironmental parameters, changes in substrate concentration and pH[8]. Other important proteins related to the metabolism of tumor cells are glucose transporter-1 (GLUT-1), the main transporter involved in glucose influx, and lactate dehydrogenase-5 (LDH-5), responsible for the conversion of pyruvate into lactate. Like MCT4, these proteins are upregulated under hypoxic conditions by HIF-1[10]. Another main target for HIF-1 is carbonic anhydrase IX (CAIX), a hypoxia-related protein involved in pH regulation[11], that shows weak correlations with the exogenous hypoxia marker pimonidazole[12,13]. The advantage of the use of these proteins as endogenous immunohistochemical markers is that no prior infusion of markers is necessary and therefore archived material can be used to assess the metabolic and, possibly, the hypoxic status of the tumor. However, up until now no endogenous marker has been identified that correlates strongly with pimonidazole[14]. In this study, we describe and quantify the expression patterns and colocalization of several important hypoxia-related and metabolic markers in biopsies of head and neck tumors and in particular the association with pimonidazole as the reference exogenous hypoxic marker[15].

## Methods

### Samples

The study was approved by the local ethics committee. 20 biopsies from 18 head and neck tumors were included in the analysis; from two tumors two biopsies were available. All patients received pimonidazole (1-((2-hydroxy-3-piperidinyl)propyl)-2-nitroimidazole hydrochloride, Hypoxyprobe-1; Natural Pharmacia International, Belmont, MA) intravenously (500 mg/m^2 ^) two hours before biopsy taking. Pimonidazole is a bioreductive chemical probe that forms protein adducts in viable hypoxic cells. Biopsies were snap frozen in liquid nitrogen and stored until further processing. The samples were cut in sections of 5 μm and stained by immunofluorescence for pimonidazole, HIF-1α, CAIX, GLUT-1, LDH-5, MCT1, MCT4 and vessels in different combinations of 3 markers per tissue section.

### Immunohistochemistry

For immunohistochemical processing, the sections were fixed for 10 minutes in acetone and rehydrated in PBS 0.1 mol/L (pH 7.4) (Klinipath, Duiven, The Netherlands). Between all consecutive steps of the staining procedure the sections were rinsed thrice for 5 minutes in PBS.

For detection of pimonidazole, sections were incubated with rabbit-anti-pimo antibody (J.A. Raleigh, Department of Radiation Oncology and Toxicology, University of North Carolina, Chapel Hill, North Carolina, USA) diluted 1:1000 in primary antibody diluent (PAD, Abcam, Cambridge, UK) for 30 minutes at 37°C. The second incubation step was with donkey-anti-rabbit Alexa488 (Molecular Probes, Leiden, The Netherlands) diluted 1:600 in PBS. Staining for vessels was done by incubation with the mouse antibody PAL-E (Euro Diagnostica, Arnhem, The Netherlands) diluted 1:10 in PAD, followed by incubation with chicken-anti-mouse Alexa647 (Molecular Probes) for 60 min at 37°C diluted 1:100 in PBS.

The same secondary antibody (in a different tissue section) was used to detect CAIX, after incubation with mouse-anti-CAIX antibody (E. Oosterwijk, Department of Urology, University Medical Center, Nijmegen), diluted 1:25 in PAD for 30 min at 37°C.

For detection of MCT1, sections were incubated with goat-anti-MCT1 (Santa Cruz)[7], 1:100 in PAD, overnight at 4°C. The next day the secondary antibody was added: donkey-anti-goatCy3 (Jackson Immuno Research Laboratories Inc., West Grove, PA, USA) 1:600 in PBS, 30 min at 37°C.

Staining for MCT4 was done by incubation with rabbit-anti-MCT4 (Santa Cruz)[16], 1:100 in PAD, overnight at 4°C. The secondary antibody was either goat-anti-rabbitFabCy3 (1:600 in PBS) or donkey-anti-rabbitAlexa488 (1:600 in PBS) depending on the combination of markers stained in that tissue section.

Sections were incubated with rabbit-anti-GLUT-1 (Neomarkers, Fremont, CA, USA.), diluted 1:100 in PAD for 30 min at 37°C, followed by donkey-anti-rabbitAlexa488, 1:600 in PBS for 30 min at 37°C for detection of GLUT-1.

Staining for HIF-1α was done by incubation with rabbit-anti-HIF-1α (Santa Cruz), diluted 1:50 in PAD overnight at 4°C, followed by goat-anti-rabbitCy3 (Jackson Immuno Research Laboratories Inc.), 1:600 in PBS for 30 min at 37°C. In the same section, all nuclei were stained with Hoechst (Sigma, Zwijndrecht), 0.33 μg/ml, in PBS for 5 min at room temperature.

Finally, for detection of LDH-5 sections were incubated with sheep-anti-LDH-5 (Abcam), diluted 1:100 in PAD overnight at 4°C. The next day, incubation with donkey-anti-sheepCy3 (Jackson Immuno Research Laboratories Inc.), 1:600 in PBS for 30 min at 37°C, completed the staining.

All sections were mounted in fluorostab (ProGen Biotechnik GmbH, Heidelberg, Germany).

### Image acquisition and analysis

The tissue sections were scanned using a digital image processing system consisting of a high-resolution 12-bit CCD camera (Micromax, Roper Scientific Inc., Trenton, NJ, USA) on a fluorescence microscope (Axioskop, Zeiss, Göttingen, Germany) and a computer-controlled motorised stepping stage. Image processing was done using IPLab software (Scanalytics Inc., Fairfax, VA, USA) on a Macintosh computer[17]. Each tissue section was sequentially scanned for the three signals at 100× magnification with a resolution of 2.6 μm/pixel. The resulting composite grey scale images were converted to binary images for further analysis. Thresholds for the fluorescent signals were interactively set above the background for each individual marker. The composite grey scale images were superimposed into one pseudocolored image for visual evaluation.

Guided by an H&E stained consecutive section, the tumor area of each section was delineated. This area was subsequently used as a mask in further analysis from which non-tumor tissue, large necrotic areas and artefacts were excluded. The marker fractions were defined as the tumor area positive for the marker, divided by the total tumor area. With this method, an automated quantitative analysis of the percentage of positively stained tumor tissue can be obtained. To determine the colocalization of the various markers with pimonidazole, the relative area positive within and outside the pimonidazole stained area was calculated. Similar analyses were done to assess the colocalization between MCT1, MCT4, GLUT-1 and CAIX.

The spatial distribution of the markers in relation to the blood vessels was measured by calculating the relative area positive in six zones around the closest vessels with a width of 50 μm each (0-50 μm, 50-100 μm, 100-150 μm, 150-200 μm, 200-250 μm and >250 μm)[17]. As the absolute fractions differ greatly, normalisation of fractions was performed for clear comparison of distributions. Quantitative analysis of the HIF-1α staining was not possible because of the low signal-to-background ratio in many HIF-1α-positive areas. All sections were visually scored by two observers and divided in two groups; high and low HIF-1α expression based on the intensity and the estimated fraction of HIF-1α positive cells. Differences between the observers were resolved at the microscope.

### Statistics

Statistical analyses were done with Prism software package for Macintosh. The relationships between metabolic parameters (as continuous variables) were tested using the Spearman rank correlation coefficient or the Pearson correlation coefficient as appropriate. Differences in colocalization were analyzed with a Mann-Whitney test. To test the association of the markers with categorical tumor characteristics (T-stage, N-stage and histopathological grade) either the Mann-Whitney test or Fisher's exact test (after dichotomization of the variables) was used.

## Results

20 biopsies of 18 patients with histologically confirmed advanced stage squamous cell carcinoma of the head and neck were included in this study. Tumor characteristics are shown in Table 1.

**Table 1:** Tumour site, stage and grade

Characteristics	Number
*Tumour site*	
Larynx	7
Hypopharynx	4
Oropharynx	5
Oral cavity	2
*T stage*	
T2	3
T3	6
T4	9
*N stage*	
N0	6
N+	12
*Histopathological grade*	
Moderately differentiated	14
Poorly differentiated	4

### Correlation between metabolic markers and tumor characteristics

**Figure 1:**
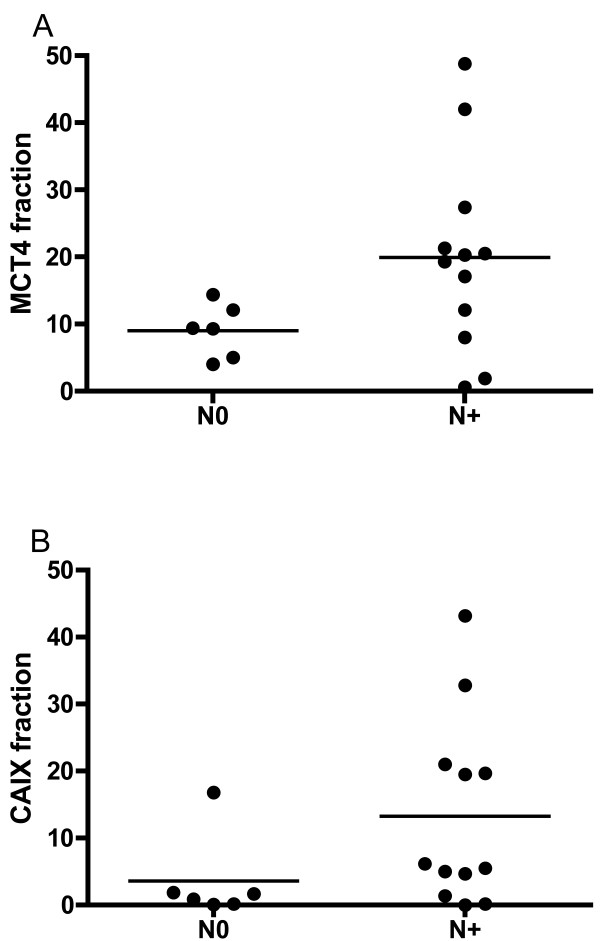
**MCT4 and CAIX expression according to nodal stage**. A significantly higher expression of MCT4 (p = 0.04, a) and CAIX (p = 0.05, b) was found in node-positive tumours. One tumor without nodal metastasis had a high CAIX expression of 17%. Interestingly, no HIF-1α expression was found in this tumor.

A trend to a higher expression of both MCT4 and CAIX in node positive patients was observed (p = 0.09 both) (Figure 1). After dichotomization of the variables MCT4 expression was significantly higher in node positive patients (p = 0.01). HIF-1α only showed a trend towards higher expression in node positive patients (p = 0.06), although HIF-1α expression was only found in one node negative tumor. In this biopsy the HIF-1α expression as well as the pimonidazole staining was found in well-differentiated areas around keratinization. T4 tumors showed a significantly lower pimonidazole staining (p = 0.02) and CAIX expression (p = 0.03) than T2 or T3 tumors. This inverse trend was found for MCT1 and MCT4 as well (p = 0.09 and p = 0.08 respectively). No correlations were found between any of the markers and differentiation grade.

### Staining patterns

**Figure 2:**
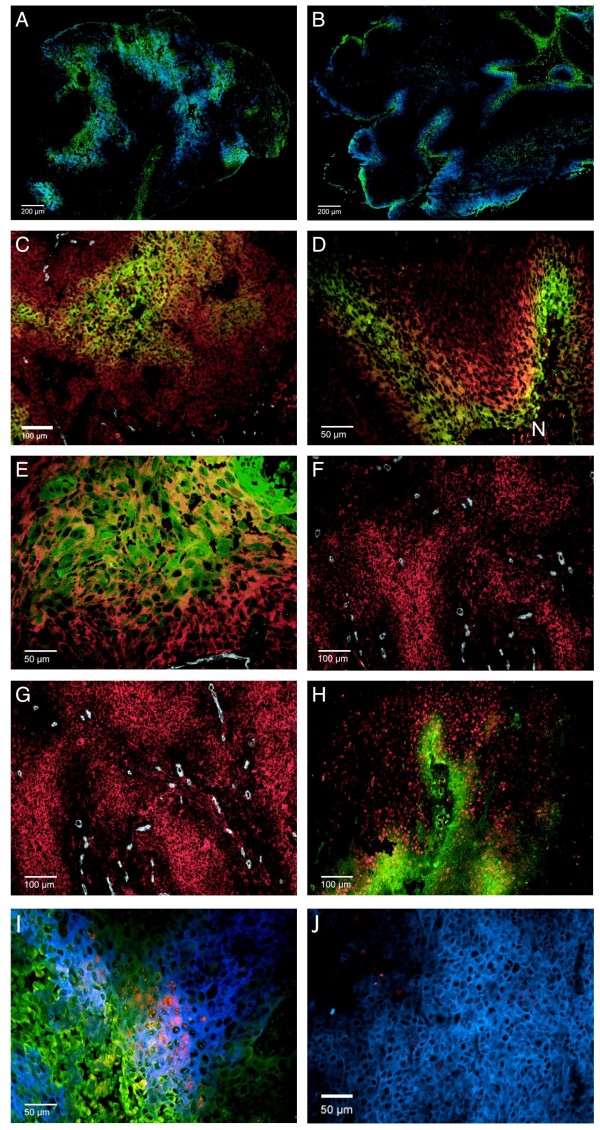
**Immunofluorescent staining pattern of metabolic and hypoxic markers**. (a and b) Fluorescent microscopic images of two head and neck carcinomas with different pimonidazole (green) and CAIX (blue) staining patterns. (c - h) Examples of staining of the various markers. (c) LDH-5 (red), pimonidazole (green) and vessels (white). (d) Perinecrotic pimonidazole (green) and CAIX (red) staining, N = Necrosis. (e) MCT1 (red), pimonidazole (green) and vessels (white), note the co-staining of MCT1 with pimonidazole. (f and g) Membranous GLUT-1 (f) and MCT4 (g) (both red) staining in relation to the vessels (white). (h) Nuclear HIF-1α (red) expression in relation to pimoniazole (green) staining. (i and j) Fluorescent microscopic images showing intense HIF-1α (red) staining in a pimonidazole-related (green) CAIX (blue) area and almost absent HIF-1α staining in another CAIX area in the same tissue section.

Examples of staining of all markers are shown in Figure 2. Pimonidazole fraction ranged from 0 - 34% (median 7%) and different hypoxic patterns were observed. Some tumors showed a typical ribbon-like pattern, others a more patchy pattern[18]. (Figure 2a/b) Extensive cytoplasmic LDH-5 expression was seen in most of the tumors (fraction range 17 - 91%, median 37%, figure 2c), with more intense staining in hypoxic areas.

Membranous CAIX expression closely followed the hypoxic pattern in some tumors, in others CAIX staining showed less spatial correlation with pimonidazole. The fraction ranged from 0 - 43% (median 4.7%) (Figure 2a, b, d).

MCT 1 showed a clear membranous staining pattern with substantial variation in intensity and extent; in some sections large MCT1 positive areas were seen, in other sections almost no MCT1 was present (fraction range 0.1 - 64%, median 14%). MCT1 staining was not only observed adjacent to hypoxic areas, but also within hypoxic areas (Figure 2e).

MCT 4 showed a more diffuse staining pattern, with fractions varying from 0.6 - 49% (median 14%) and with more staining at increasing distance from the vessels. GLUT-1 had a comparable staining pattern to MCT4 (fraction range 3 - 43%, median 18%): diffuse staining throughout the tissue section, with more staining at increasing distance from the vessels. (Figure 2f/g)

Apparent localised nuclear HIF-1α staining was present in 15 out of 20 biopsies, mostly in and around hypoxic areas (Figure 2h) with large variation in intensity. In some tissue sections membranous or cytoplasmatic staining was present as well, but only nuclear staining was taken into account.

### Correlations between markers

**Figure 3:**
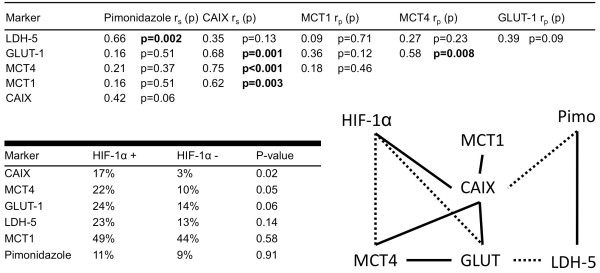
**Correlations between the endogenous markers and pimonidazole**. A schematic representation has been added; solid lines represent a significant correlation, dashed lines a trend (p-value between 0.05-0.1). Percentages in the second table are mean values.

To get a global impression of the associations between the six markers overall correlations were calculated, irrespective of the geographical distribution. (Figure 3) The strongest correlation with pimonidazole was observed for LDH-5 fraction (r = 0.66, p = 0.002). CAIX showed significant correlations with MCT1, MCT4 and GLUT-1.

The expression of the endogenous markers was compared between biopsies with a high and low HIF-1α expression. (Figure 3) Overall, biopsies with a high HIF-1α expression demonstrated a significantly higher CAIX fraction. Remarkably, two biopsies clearly showed HIF-1α expression, but very low fractions of CAIX, GLUT-1 and MCT4. In one of the biopsies pimonidazole staining was associated with well-differentiated keratinizing areas as described by Janssen et al. [19], the other biopsy showed no pimonidazole staining at all. Overall, mean pimonidazole staining was equal in biopsies with high and low HIF-1α expression. A schematic representation of the associations between the markers is shown in Figure 3.

### Colocalization of metabolic and hypoxic markers

**Figure 4:**
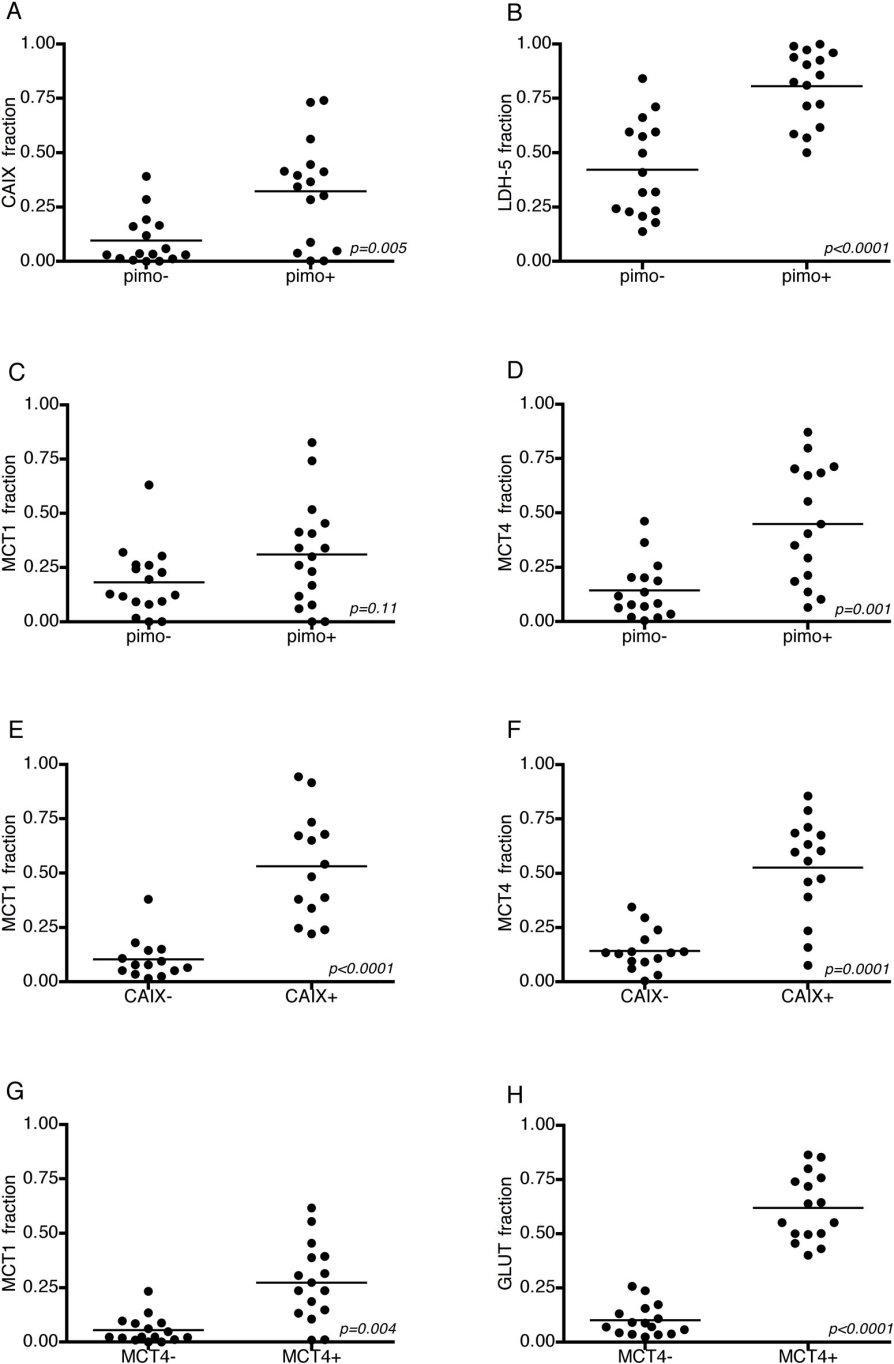
**Colocalization of the various markers**. (a-d) CAIX, LDH-5 and MCT4 show a significantly higher expression in pimonidazole stained areas, MCT1 expression is not significantly different. (e and f) Expression of MCT1 and MCT4 in CAIX negative and positive areas, both are significantly higher in areas expressing CAIX. (g and h) Increased expression of MCT1 and GLUT in MCT4 positive areas.

The results of the quantitative analysis of the colocalization of the various markers are shown in Figure 4. MCT4 expression was significantly higher in hypoxic areas than in non-hypoxic areas (p = 0.001) and also clearly correlated to CAIX expression. GLUT-1 expression and MCT4 expression showed an even stronger amount of colocalization with approximately six times higher GLUT-1 expression in MCT4 positive areas.

MCT1 was present in well-oxygenated areas, but unexpectedly showed considerable expression in hypoxic areas as well (no significant difference, p = 0.11). Additionally, MCT1 expression was significantly correlated with CAIX expression (p < 0.001). Pimonidazole stained areas exhibited, beside MCT4, a significantly higher expression of CAIX and LDH-5 compared to pimonidazole negative areas.

Although colocalization with HIF-1α expression could not be analyzed in the same manner as the other markers, it was observed that pimonidazole-related CAIX areas showed more intense HIF-1α expression than CAIX areas far from pimonidazole positive hypoxic areas (Figure 2i-j).

### Relationship of markers with vasculature

**Figure 5:**
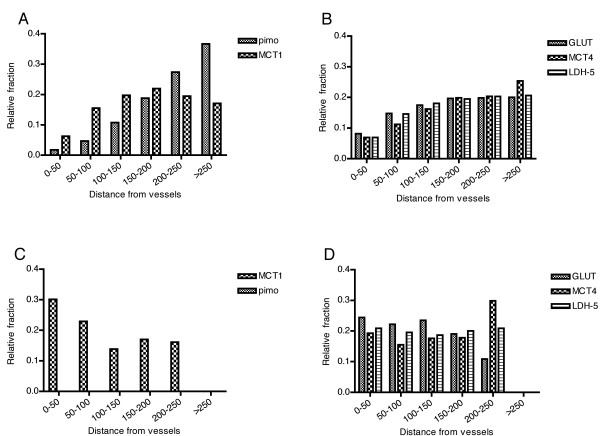
**Marker expression in different zones around the vessels**. Zone analysis in a biopsy with a large hypoxic fraction (upper panels) and a tumor with no pimonidazole staining (lower panels). Pimonidazole staining increases steadily at larger distances from the vessels (a). The increase of GLUT, MCT4 and LDH-5 expression is less steep with a plateau reached between 150-200 μm (b). MCT1 expression increases until 150-200 μm, a slight decrease is noted at larger distances (a). In the tumour with no pimonidazole staining, a decrease in MCT1 expression (c) and no increase of GLUT, MCT4 and LDH-5 expression (d) is observed at larger distances from the vessels.

Chronic hypoxia is an important feature of malignant tumors, marked by a tissue oxygen gradient with lower oxygen tensions farther from the blood vessels. The expression of the endogenous markers in relation to the vessels, with pimonidazole as a reference hypoxic marker, can provide valuable information about their usefulness as a marker of chronic hypoxia. To assess the expression of the markers in relation to the vessels, fractions were calculated at different distances from the most nearby vessel in steps of 50 μm. In general, hypoxic fraction and LDH-5 staining increased at larger distances from the vessels. GLUT-1 and MCT4 were present in variable fractions in the proximity of vessels, but the expression increased about two to four-fold at > 150 μm from the vessels. All except four tumors exhibited this "chronic hypoxic pattern". These remaining four showed no pimonidazole staining. Two examples of the distribution of the markers in tumors with pimonidazole (chronic hypoxic pattern) and without pimonidazole staining (non-hypoxic pattern) are shown in Figure 5. In one tumor (5a and b) with a large hypoxic fraction (20%) pimonidazole binding increases steeply with distance from the vessels. GLUT-1, MCT4 and LDH-5 follow this pattern but less steep, with a plateau reached between 150-200 μm. MCT1 expression increases until 150-200 μm and decreases at larger distances. In the second tumor (Figure 5c and 5d) all metabolic markers were strongly expressed without any relationship with the vessels and, in the absence of pimonidazole, not depending on hypoxia. Low HIF-1α and CAIX expression was present in this tumor.

MCT1 expression clearly showed a different pattern compared to the other markers; in most tumors the MCT1 fraction increased slightly till 100 μm from the vessels and remained constant or modestly decreased again at larger distances. However, in the four tumors without hypoxia, MCT1 expression was present close to the vessels and decreased farther from the vessels.

## Discussion

Hypoxia is an important feature of advanced head and neck tumors, with a negative influence on prognosis[12,20]. Evaluation of cellular responses to hypoxia can be of clinical relevance in a prognostic and predictive way and possibly for treatment adaptation. Until now no endogenous marker has been found that strongly and consistently correlates with hypoxia, so different kind of analyses as presented here are crucial to elucidate the role of the various proteins and the response of tumor cells to a hypoxic microenvironment.

Although a global analysis of the overall expression of a certain protein allows easy comparison between biopsies and between different studies, it is a huge simplification of the true heterogeneous situation in a tumor biopsy. Therefore, to gain more insight in the spatial relationship between the metabolic markers and the oxygenation status in the tumor, we additionally assessed the expression of the proteins within and outside pimonidazole stained areas and the relation to the vessels. With this analysis more markers show an evident relationship with pimonidazole than with a global correlation analysis (Figure 4).

Of the endogenous hypoxia-related markers that we examined, LDH-5 has the strongest relationship with pimonidazole. LDH-5 is one of the target enzymes of HIF-1 and has been described to have a strong association with HIF-1α expression in tumor tissue sections [21,22]. The high expression of LDH-5 in hypoxic areas is as expected; it is one of the key enzymes in the glycolysis, the primary energy source in the absence of oxygen. The reason for a considerable expression of LDH-5 outside hypoxic areas could be the high rate of aerobe glycolysis (the Warburg effect) as often observed in malignant tumors[4], although alternative explanations are possible, as pyruvate can originate from other pathways as well[23]. It should be noted that due to the similarity between LDH-5 and the other LDH isoforms, there is a possibility of some cross-reactivity of the antibody.

CAIX is previously described to correlate weakly with pimonidazole binding[13,24,25]. Despite the weak overall correlation, CAIX fraction was significantly higher in the hypoxic (pimonidazole positive) areas. This emphasizes the strong association of this protein with hypoxia that could not be found by a simple correlation analysis. Interestingly, although all the endogenous markers, with the exception of MCT1, are regulated by HIF-1[9,10], the staining patterns and colocalization differ. An interesting observation is the strong resemblance of the HIF-1α and CAIX staining pattern (intermediate hypoxic areas). This typical pattern circumferences the pimonidazole stained area with a partial overlap (Figure 2i). The colocalization of CAIX with HIF-1α is far from perfect, but more concordant than that of GLUT-1, MCT4 and LDH-5 with HIF-1α. The strong relationship of CAIX with HIF-1 is known, with CAIX expression tightly controlled by HIF-1[26]. In this context, an interesting observation is the higher level of HIF-1α expression in hypoxia-related CAIX areas than in CAIX areas separate from hypoxic (pimonidazole stained) areas (Figure 2i/j). A possible explanation of these findings could be the increased transcription of HIF-1α without stabilization in the intermediate hypoxic areas (areas with CAIX expression without HIF-1α staining) and stabilization of HIF-1α in severe hypoxic areas (areas overlapping and adjacent to pimonidazole with CAIX and HIF-1α staining)[27]. It could also indicate the transient presence of acute hypoxic areas, which are reoxygenated before pimonidazole administration. It was shown in SiHa (human cervical squamous cell carcinoma) tumors that up to 20% of the tumor cells were intermittently hypoxic over an 8-hour period[28]. This explanation is less likely as CAIX upregulation requires a longer period of hypoxia[29]. A third explanation could be the induction of HIF-1α and consequent activation of CAIX through a hypoxia-independent mechanism without stabilization of HIF-1α under these conditions. An acidic tumor microenvironment can influence HIF-1α and CAIX expression as well[29,30]. In any case, there is little evidence for a HIF-1 independent CAIX activating mechanism[26].

Most markers showed a range of co-expression, but a very strong spatial relationship was found between GLUT-1 and MCT4 (Figure 4h). These showed a comparable, typical expression increasing at larger distances from the vessels, with a two- to four-fold increase of the fraction at 150-200 μm. This pattern and agreement reflects their interrelated role in the glycolytic pathway under hypoxic conditions, GLUT-1 for glucose import and MCT4 for lactate export. LDH-5, one of the intermediary enzymes in this pathway, shows a similar pattern, but was not stained in the same section as MCT4 or GLUT-1, so the amount of colocalization could not be calculated.

Although a small series, a positive correlation between MCT4 and N-stage was found. Overexpression of MCT4 in malignancies has been described in colorectal cancer[31] and cervical carcinomas[32]. Except for a trend towards shorter overall survival with MCT4 positive adenosquamous carcinomas of the cervix, no correlations with clinicopathological data have been found before. These findings indicate that MCT4 is a potential marker for the aggressiveness of a tumor.

MCT1 expression in > 5% of the tumor area was present in 14 of the 20 biopsies. MCT1 expression in tumors has been described in lung cancer[33], brain tumors[34] and cervical cancer[32]. However, in the colonic epithelium, Lambert et al. found a decline in expression associated with transition to malignancy[35]. In our study, MCT1 expression was present in oxic as well as hypoxic areas, in contrast to the observation made by Sonveaux et al. in biopsies of lung carcinomas[6]. It is important to note that in our study a different antibody was used of which some aspecificity formally can not be excluded, although other studies show good results with this antibody as well[7,36]. As the fraction MCT1 even increased at larger distance from the vessels, it seems likely that MCT1, beside MCT4, plays a role in lactate export in hypoxic areas. It is reasonable to assume that in tumor cells, like in red blood cells, depending on the substrate concentrations and pH, MCT1 functions as either a lactate importer or assists MCT4 in lactate export[37]. Either way, MCT1 has already shown some potential as a therapeutic target in vitro. Inhibition of MCT1 by lonidamine induced a strong decrease in intracellular pH and loss of viability of the tumor cells[34]. The MCT1 inhibitor α-cyano-4-hydroxycinnamate blocks lactate-fueled respiration in tumor cells and induces tumor growth retardation in a mouse model[6]. Of interest is the strong correlation and colocalization of MCT1 with CAIX (Figure 3 and 4e), affirming an important role in pH regulation as described in melanoma and neuroblastoma[34,38].

In conclusion, metabolic markers show a strong but irregular relation with hypoxia with obvious correlations between markers, emphasizing the complex metabolic regulatory system with a strong environmental (hypoxia, pH) influence. Co-expression of markers provides additional information over single marker fractions. MCT4 and GLUT-1 show a typical "diffusion-limited hypoxic" pattern with a strong colocalization indicating activation by similar stimuli. The positive correlation with N-stage makes MCT4 a potential marker for tumor aggressiveness, but its exact value still has to be established. MCT1 overexpression is present in the majority of the advanced head and neck carcinomas in our series, in non-hypoxic as well as hypoxic areas. The strong colocalization with CAIX suggests an important role in pH regulation.

## Conclusion

Endogenous metabolic and hypoxia-related markers can be of great importance as prognostic and predictive markers and are potential therapeutic targets. As the various markers respond differently to hypoxia and other environmental factors, a combination of these markers could be used to predict treatment outcome and select the appropriate patients for new targeted therapies.

## Competing interests

The authors declare that they have no competing interests.

## Authors' contributions

SR designed the study, participated in the acquisition of data, analyzed the data and drafted the manuscript. JL participated in the acquisition of data. AK contributed to the design of the study. JB contributed to the interpretation of data. HK contributed to the design of the study and the interpretation of data. All authors critically revised the manuscript and approved the final version.

## Pre-publication history

The pre-publication history for this paper can be accessed here:

http://www.biomedcentral.com/1471-2407/11/167/prepub

## References

[B1] Vander Heiden MG Cantley LC Thompson CB Understanding the Warburg effect: the metabolic requirements of cell proliferation Science 2009 324 1029 1033 10.1126/science.1160809 19460998 PMC2849637

[B2] Porter JR Louis PASTEUR; achievements and disappointments, 1861 Bacteriol Rev 1961 25 389 403 14037390 10.1128/br.25.4.389-403.1961 PMC441122

[B3] Warburg O Wind F Negelein E The Metabolism of Tumors in the Body J Gen Physiol 1927 8 519 530 10.1085/jgp.8.6.519 19872213 PMC2140820

[B4] Busk M Horsman MR Kristjansen PE van der Kogel AJ Bussink J Overgaard J Aerobic glycolysis in cancers: implications for the usability of oxygen-responsive genes and fluorodeoxyglucose-PET as markers of tissue hypoxia Int J Cancer 2008 122 2726 2734 10.1002/ijc.23449 18351643

[B5] Kim JW Dang CV Cancer's molecular sweet tooth and the Warburg effect Cancer Res 2006 66 8927 8930 10.1158/0008-5472.CAN-06-1501 16982728

[B6] Sonveaux P Vegran F Schroeder T Wergin MC Verrax J Rabbani ZN De Saedeleer CJ Kennedy KM Diepart C Jordan BF Kelley MJ Gallez B Wahl ML Feron O Dewhirst MW Targeting lactate-fueled respiration selectively kills hypoxic tumor cells in mice J Clin Invest 2008 118 3930 3942 19033663 10.1172/JCI36843 PMC2582933

[B7] Koukourakis MI Giatromanolaki A Harris AL Sivridis E Comparison of metabolic pathways between cancer cells and stromal cells in colorectal carcinomas: a metabolic survival role for tumor-associated stroma Cancer Res 2006 66 632 637 10.1158/0008-5472.CAN-05-3260 16423989

[B8] Enerson BE Drewes LR Molecular features, regulation, and function of monocarboxylate transporters: implications for drug delivery J Pharm Sci 2003 92 1531 1544 10.1002/jps.10389 12884241

[B9] Ullah MS Davies AJ Halestrap AP The plasma membrane lactate transporter MCT4, but not MCT1, is up-regulated by hypoxia through a HIF-1alpha-dependent mechanism J Biol Chem 2006 281 9030 9037 16452478 10.1074/jbc.M511397200

[B10] Semenza GL Regulation of cancer cell metabolism by hypoxia-inducible factor 1 Semin Cancer Biol 2009 19 12 16 10.1016/j.semcancer.2008.11.009 19114105

[B11] Potter CP Harris AL Diagnostic, prognostic and therapeutic implications of carbonic anhydrases in cancer Br J Cancer 2003 89 2 7 10.1038/sj.bjc.6600936 12838292 PMC2394207

[B12] Kaanders JHAM Wijffels KIEM Marres HAM Ljungkvist ASE Pop LAM van den Hoogen FJA de Wilde PCM Bussink J Raleigh JA van der Kogel AJ Pimonidazole binding and tumor vascularity predict for treatment outcome in head and neck cancer Cancer Res 2002 62 7066 7074 12460928

[B13] Hoogsteen IJ Lok J Marres HA Takes RP Rijken PF van der Kogel AJ Kaanders JH Hypoxia in larynx carcinomas assessed by pimonidazole binding and the value of CA-IX and vascularity as surrogate markers of hypoxia Eur J Cancer 2009 45 2906 2914 10.1016/j.ejca.2009.07.012 19699082

[B14] Rademakers SE Span PN Kaanders JH Sweep FC van der Kogel AJ Bussink J Molecular aspects of tumour hypoxia Mol Oncol 2008 2 41 53 10.1016/j.molonc.2008.03.006 19383328 PMC5527797

[B15] Raleigh JA Chou SC Arteel GE Horsman MR Comparisons among pimonidazole binding, oxygen electrode measurements, and radiation response in C3H mouse tumors Radiat Res 1999 151 580 589 10.2307/3580034 10319731

[B16] Schmidt S Richter M Montag D Sartorius T Gawlik V Hennige AM Scherneck S Himmelbauer H Lutz SZ Augustin R Kluge R Ruth P Joost HG Schurmann A Neuronal functions, feeding behavior, and energy balance in Slc2a3+/- mice Am J Physiol Endocrinol Metab 2008 295 1084 1094 10.1152/ajpendo.90491.2008 18780771

[B17] Rijken PF Bernsen HJ Peters JP Hodgkiss RJ Raleigh JA van der Kogel AJ Spatial relationship between hypoxia and the (perfused) vascular network in a human glioma xenograft: a quantitative multi-parameter analysis Int J Radiat Oncol Biol Phys 2000 48 571 582 10.1016/S0360-3016(00)00686-6 10974478

[B18] Ljungkvist AS Bussink J Rijken PF Kaanders JH van der Kogel AJ Denekamp J Vascular architecture, hypoxia, and proliferation in first-generation xenografts of human head-and-neck squamous cell carcinomas Int J Radiat Oncol Biol Phys 2002 54 215 228 12182995 10.1016/s0360-3016(02)02938-3

[B19] Janssen HL Hoebers FJ Sprong D Goethals L Williams KJ Stratford IJ Haustermans KM Balm AJ Begg AC Differentiation-associated staining with anti-pimonidazole antibodies in head and neck tumors Radiother Oncol 2004 70 91 97 10.1016/j.radonc.2003.09.012 15036858

[B20] Nordsmark M Bentzen SM Rudat V Brizel D Lartigau E Stadler P Becker A Adam M Molls M Dunst J Terris DJ Overgaard J Prognostic value of tumor oxygenation in 397 head and neck tumors after primary radiation therapy. An international multi-center study Radiother Oncol 2005 77 18 24 10.1016/j.radonc.2005.06.038 16098619

[B21] Koukourakis MI Giatromanolaki A Winter S Leek R Sivridis E Harris AL Lactate dehydrogenase 5 expression in squamous cell head and neck cancer relates to prognosis following radical or postoperative radiotherapy Oncology 2009 77 285 292 10.1159/000259260 19923867

[B22] Koukourakis MI Giatromanolaki A Sivridis E Gatter KC Harris AL Lactate dehydrogenase 5 expression in operable colorectal cancer: strong association with survival and activated vascular endothelial growth factor pathway--a report of the Tumour Angiogenesis Research Group J Clin Oncol 2006 24 4301 4308 10.1200/JCO.2006.05.9501 16896001

[B23] DeBerardinis RJ Cheng T Q's next: the diverse functions of glutamine in metabolism, cell biology and cancer Oncogene 2010 29 313 324 10.1038/onc.2009.358 19881548 PMC2809806

[B24] Olive PL Aquino-Parsons C MacPhail SH Liao SY Raleigh JA Lerman MI Stanbridge EJ Carbonic anhydrase 9 as an endogenous marker for hypoxic cells in cervical cancer Cancer Res 2001 61 8924 8929 11751418

[B25] Airley RE Loncaster J Raleigh JA Harris AL Davidson SE Hunter RD West CM Stratford IJ GLUT-1 and CAIX as intrinsic markers of hypoxia in carcinoma of the cervix: relationship to pimonidazole binding Int J Cancer 2003 104 85 91 10.1002/ijc.10904 12532423

[B26] Kaluz S Kaluzova M Liao SY Lerman M Stanbridge EJ Transcriptional control of the tumor- and hypoxia-marker carbonic anhydrase 9: A one transcription factor (HIF-1) show? Biochim Biophys Acta 2009 1795 162 172 19344680 10.1016/j.bbcan.2009.01.001 PMC2670353

[B27] Kaluz S Kaluzova M Chrastina A Olive PL Pastorekova S Pastorek J Lerman MI Stanbridge EJ Lowered oxygen tension induces expression of the hypoxia marker MN/carbonic anhydrase IX in the absence of hypoxia-inducible factor 1 alpha stabilization: a role for phosphatidylinositol 3'-kinase Cancer Res 2002 62 4469 4477 12154057

[B28] Bennewith KL Durand RE Quantifying transient hypoxia in human tumor xenografts by flow cytometry Cancer Res 2004 64 6183 6189 10.1158/0008-5472.CAN-04-0289 15342403

[B29] Sorensen BS Alsner J Overgaard J Horsman MR Hypoxia induced expression of endogenous markers in vitro is highly influenced by pH Radiother Oncol 2007 83 362 366 10.1016/j.radonc.2007.04.028 17512623

[B30] Mekhail K Gunaratnam L Bonicalzi ME Lee S HIF activation by pH-dependent nucleolar sequestration of VHL Nat Cell Biol 2004 6 642 647 10.1038/ncb1144 15181450

[B31] Pinheiro C Longatto-Filho A Scapulatempo C Ferreira L Martins S Pellerin L Rodrigues M Alves VA Schmitt F Baltazar F Increased expression of monocarboxylate transporters 1, 2, and 4 in colorectal carcinomas Virchows Arch 2008 452 139 146 10.1007/s00428-007-0558-5 18188595

[B32] Pinheiro C Longatto-Filho A Ferreira L Pereira SM Etlinger D Moreira MA Jube LF Queiroz GS Schmitt F Baltazar F Increasing expression of monocarboxylate transporters 1 and 4 along progression to invasive cervical carcinoma Int J Gynecol Pathol 2008 27 568 574 10.1097/PGP.0b013e31817b5b40 18753962

[B33] Koukourakis MI Giatromanolaki A Bougioukas G Sivridis E Lung cancer: a comparative study of metabolism related protein expression in cancer cells and tumor associated stroma Cancer Biol Ther 2007 6 1476 1479 10.4161/cbt.6.9.4635 17881895

[B34] Fang J Quinones QJ Holman TL Morowitz MJ Wang Q Zhao H Sivo F Maris JM Wahl ML The H+-linked monocarboxylate transporter (MCT1/SLC16A1): a potential therapeutic target for high-risk neuroblastoma Mol Pharmacol 2006 70 2108 2115 10.1124/mol.106.026245 17000864

[B35] Lambert DW Wood IS Ellis A Shirazi-Beechey SP Molecular changes in the expression of human colonic nutrient transporters during the transition from normality to malignancy Br J Cancer 2002 86 1262 1269 10.1038/sj.bjc.6600264 11953883 PMC2375337

[B36] Thibault R De Coppet P Daly K Bourreille A Cuff M Bonnet C Mosnier JF Galmiche JP Shirazi-Beechey S Segain JP Down-regulation of the monocarboxylate transporter 1 is involved in butyrate deficiency during intestinal inflammation Gastroenterology 2002 133 1916 1927 10.1053/j.gastro.2007.08.041 18054563

[B37] Halestrap AP Price NT The proton-linked monocarboxylate transporter (MCT) family: structure, function and regulation Biochem J 1999 343 Pt 2 281 299 10510291 PMC1220552

[B38] Wahl ML Owen JA Burd R Herlands RA Nogami SS Rodeck U Berd D Leeper DB Owen CS Regulation of intracellular pH in human melanoma: potential therapeutic implications Mol Cancer Ther 2002 1 617 628 12479222

